# *Notes from the Field:* First Reported U.S. Cases of Tinea Caused by *Trichophyton indotineae —* New York City, December 2021–March 2023

**DOI:** 10.15585/mmwr.mm7219a4

**Published:** 2023-05-12

**Authors:** Avrom S. Caplan, Sudha Chaturvedi, YanChun Zhu, Gabrielle C. Todd, Lu Yin, Adriana Lopez, Lisa Travis, Dallas J. Smith, Tom Chiller, Shawn R. Lockhart, Karen A. Alroy, William G. Greendyke, Jeremy A. W. Gold

**Affiliations:** ^1^The Ronald O. Perelman Department of Dermatology, NYU Grossman School of Medicine, New York, New York; ^2^Wadsworth Center, New York State Department of Health; ^3^Division of Foodborne, Waterborne, and Environmental Diseases, National Center for Emerging and Zoonotic Infectious Diseases, CDC; ^4^Epidemic Intelligence Service, CDC; ^5^New York City Department of Health and Mental Hygiene, New York, New York.

Tinea is a common, highly contagious, superficial infection of the skin, hair, or nails caused by dermatophyte molds.* During the past decade, an epidemic of severe, antifungal-resistant tinea has emerged in South Asia because of the rapid spread of *Trichophyton indotineae*,^†^ a novel dermatophyte species; the epidemic has likely been driven by misuse and overuse of topical antifungals and corticosteroids^§^ (*1*,*2*). *T. indotineae* infections are highly transmissible and characterized by widespread, inflamed, pruritic plaques on the body (tinea corporis), the crural fold, pubic region, and adjacent thigh (tinea cruris), or the face (tinea faciei) (*1*). *T. indotineae* isolates are frequently resistant to terbinafine, a mainstay of tinea treatment (*1*,*3*). *T. indotineae* infections have been reported throughout Asia and in Europe and Canada but have not previously been described in the United States (*3*).

On February 28, 2023, a New York City dermatologist notified public health officials of two patients who had severe tinea that did not improve with oral terbinafine treatment, raising concern for potential *T. indotineae* infection; these patients shared no epidemiologic links. Skin culture isolates from each patient were previously identified by a clinical laboratory as *Trichophyton mentagrophytes* and were subsequently forwarded to the Wadsworth Center, New York State Department of Health, for further review and analysis. Sanger sequencing of the internal transcribed spacer region of the ribosomal gene, followed by phylogenetic analysis performed during March 2023, identified the isolates as *T. indotineae* (Supplementary Figure; https://stacks.cdc.gov/view/cdc/127678). Activity related to this investigation was reviewed by CDC and was conducted consistent with applicable federal law and CDC policy.^¶^

Patient A, a woman aged 28 years, developed a widespread pruritic eruption during summer 2021. She had a first dermatologic evaluation in December 2021, at which time she was in her third trimester of pregnancy. She had no other underlying medical conditions, no known exposures to a person with similar rash, and no recent international travel history. Dermatologists noted large, annular, scaly, pruritic plaques over the neck, abdomen, pubic region, and buttocks (Figure). She received a diagnosis of tinea and began oral terbinafine therapy in January 2022 after the birth of her baby. Because her eruptions did not improve after 2 weeks of therapy, terbinafine was discontinued, and she began itraconazole treatment. The rash resolved completely after completing a 4-week course of itraconazole; however, she is being monitored for potential recurrence of infection and the need for resumption of itraconazole.

**FIGURE:**
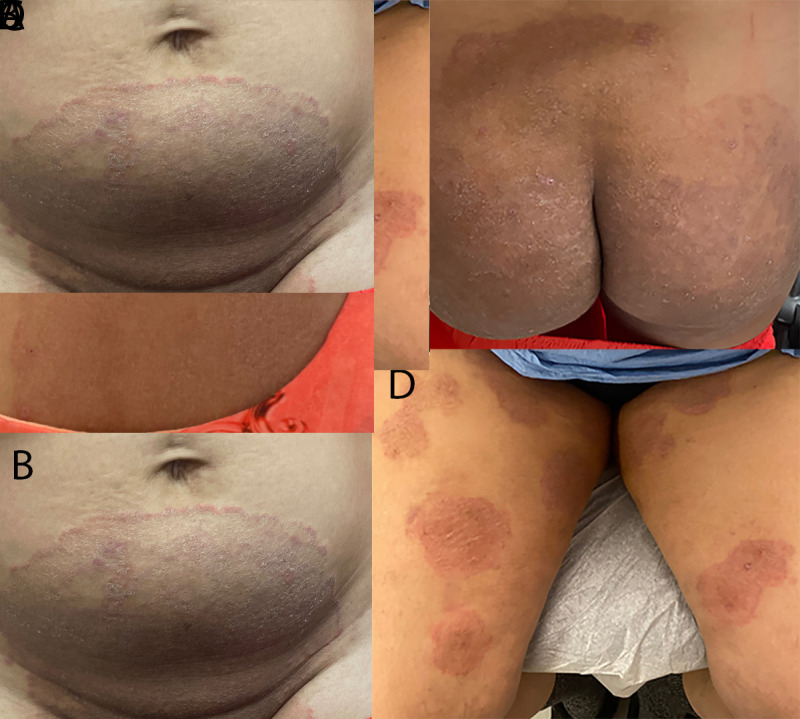
Lesions occurring on two patients with first reported U.S. cases of tinea caused by *Trichophyton indotineae*, on patient A’s neck, abdomen, and buttocks (A–C)* and on patient B’s thighs^†^ (D) — New York City, December 2021–March 2023 Photos/Lu Yin (A–C) and Vignesh Ramachandran (D). Used with patients’ permission. * At initial dermatology evaluation, patient A had large, annular, scaly, and pruritic erythematous plaques of the neck, abdomen, groin, and buttocks. ^†^ At initial dermatology evaluation, patient B had widespread, discrete, scaly, annular, pruritic plaques affecting the thighs and buttocks.

Patient B, a woman aged 47 years with no major medical conditions, developed a widespread, pruritic eruption in summer 2022 while in Bangladesh. There, she received treatment with topical antifungal and steroid combination creams and noted that several family members were experiencing similar eruptions. After returning to the United States, she visited an emergency department three times during autumn 2022. She was prescribed hydrocortisone 2.5% ointment and diphenhydramine (visit 1), clotrimazole cream (visit 2), and terbinafine cream (visit 3) with no improvement. In December 2022, she was evaluated by dermatologists who noted widespread, discrete, scaly, annular, pruritic plaques affecting the thighs and buttocks (Figure). She received a 4-week course of oral terbinafine, but her symptoms did not improve. She then received a 4-week course of griseofulvin therapy, resulting in approximately 80% improvement. Itraconazole therapy is being considered pending further evaluation given the recent confirmation of suspected *T. indotineae* infection. Her son and husband, who live in the same house and report similar eruptions, are currently undergoing evaluation.

The cases in these two patients highlight several important points. Patient A had no recent international travel history, suggesting potential local U.S. transmission of *T. indotineae*. Health care providers should consider *T. indotineae* infection in patients with widespread tinea, particularly when eruptions do not improve with first-line topical antifungal agents or oral terbinafine. Culture-based identification techniques used by most clinical laboratories typically misidentify *T. indotineae* as *T. mentographytes* or *T. interdigitale*; correct identification requires genomic sequencing. Health care providers who suspect *T. indotineae* infection should contact their state or local public health department for assistance with testing,** which is available at certain public health laboratories and specialized academic and commercial laboratories. Successful treatment using oral itraconazole, a triazole antifungal, has been documented. However, providers should be aware of challenges with itraconazole absorption,^††^ which can lead to variable serum drug concentrations; itraconazole's interactions with other drugs; the need for up to 12 weeks of therapy (*3*); and the documented emergence of triazole resistance (*4*,*5*). Antimicrobial stewardship efforts are essential to minimize the misuse and overuse of prescribed and over-the-counter antifungal drugs and corticosteroids. In addition, health care providers can educate patients about strategies to prevent the spread of the dermatophytes that cause tinea.^§§^ Finally, public health surveillance efforts and increased testing could help detect and monitor the spread of *T. indotineae*.

## Footnotes

* Commonly known as “ringworm,” tinea is most often caused by dermatophyte molds belonging to the genus *Trichophyton*. The infection spreads easily by skin-to-skin contact with infected animals or persons, secondary spread from other affected body sites, and fomites. Most skin infections are localized and resolve with topical antifungal treatment, and oral antifungal therapy is generally reserved for cases that do not improve with topical treatment or those with extensive disease or infection of the hair follicles. https://www.cdc.gov/fungal/diseases/ringworm/definition.html

† The etiologic agent causing the epidemic of drug-resistant tinea in South Asia was initially identified as *T. mentagrophytes* ITS genotype VIII. However, based on recent genomic studies, scientists determined that these frequently terbinafine-resistant *Trichophyton* strains were sufficiently different from *T. mentagrophytes* to be considered a new species, *T. indotineae*.

§ The emergence and spread of *T. indotineae* in South Asian countries have been linked to the inappropriate use of widely available topical combination creams containing antifungals, antibiotics, and high-potency corticosteroids. https://www.cdc.gov/fungal/diseases/ringworm/dermatophyte-resistance.html

¶ 45 C.F.R. part 46.102(l)(2), 21 C.F.R. part 56; 42 U.S.C. Sect. 241(d); 5 U.S.C. Sect. 552a; 44 U.S.C. Sect. 3501 et seq.

** Public health officials who are concerned about potential cases of drug-resistant tinea infections can email fungaloutbreaks@cdc.gov for assistance with recommendations and testing.

††
https://www.accessdata.fda.gov/drugsatfda_docs/label/2010/022484s000lbl.pdf

§§
https://www.cdc.gov/fungal/diseases/ringworm/risk-prevention.html
